# A Novel Bacterium, *Butyricimonas virosa*, Preventing HFD-Induced Diabetes and Metabolic Disorders in Mice *via* GLP-1 Receptor

**DOI:** 10.3389/fmicb.2022.858192

**Published:** 2022-05-17

**Authors:** Heetae Lee, Jinho An, Jiyeon Kim, Dohyun Choi, Youngcheon Song, Chong-Kil Lee, Hyunseok Kong, Sang Bum Kim, Kyungjae Kim

**Affiliations:** ^1^College of Pharmacy, Sahmyook University, Seoul, South Korea; ^2^College of Pharmacy, Chungbuk National University, Cheongju, South Korea; ^3^College of Animal Biotechnology and Resource, Sahmyook University, Seoul, South Korea

**Keywords:** *Butyricimonas virosa*, gut microbiota, GLP-1 receptor, hyperglycemia, PPARα

## Abstract

Knowledge of the impact of the gut microbiota on human health has increased, and modulation of the bacterial community is now considered a therapeutic target for various diseases. Certain novel bacterial species have probiotic properties associated with improvement in obesity and related metabolic disorders. The relative abundance of *Butyricimonas* spp. is correlated with metabolic parameters; however, the physiological role of *Butyricimonas* in metabolic improvement is unclear. In this study, live and heat-killed *Butyricimonas virosa* were administered to mice with high-fat diet (HFD)-induced obesity. Both live and heat-killed *B. virosa* ameliorated HFD-impaired body weight, serum glucose level, insulin resistance, and liver steatosis. Moreover, activation of the glucagon-like peptide-1 receptor (GLP-1R) and peroxisome proliferator-activated receptor α (PPARα) was observed in the liver, and the expression levels of insulin receptor substrate (IRS)-1, IRS-2, Toll-like receptor 5 (TLR5), and zonula occludens-1 (ZO-1) were upregulated in the ileum. Finally, we demonstrated that the effect of *B. virosa* treatment on glucose regulation may be linked to the upregulation of GLP-1R in the liver and is not a result of colonization of the gut by *B. virosa* or *B. virosa*-produced butyrate. Our results provide a rationale for the development of *Butyricimonas* spp.-based therapeutics and prophylactics for hyperglycemia.

## Introduction

The effect of the gut microbiota on human health has been investigated in the past decade. The intestinal microbial ecology is considered an important factor in energy metabolism and the immune responses to various diseases (Boulange et al., 2016). Importantly, novel microbes residing in the human gut were identified by next-generation sequencing. Certain of these microbes were named pharmabiotics because they exerted therapeutic effects beyond those of probiotics. For example, *Akkermansia muciniphila* and *Faecalibacterium prausnitzii* are considered therapeutic targets for multiple diseases (Derrien et al., 2017; Martin et al., 2017; Depommier et al., 2019). Moreover, a recent human clinical study demonstrated that *A. muciniphila* significantly improved metabolic parameters (including insulin sensitivity and total cholesterol level), such that it is considered a pharmabiotic (Depommier et al., 2019).

Microbes with therapeutic effects on metabolic disorders including obesity, hyperglycemia, and hypercholesterolemia have been reported (Okubo et al., 2018). Potential pharmabiotic candidates typically have common properties, such as the ability to produce short-chain fatty acids (SCFAs), enhance gut barrier function, improve gut microbial imbalance, and regulate inflammatory immune responses (Nguyen et al., 2017). These beneficial effects are linked to improvements in insulin resistance and lipid metabolism, as well as reduced inflammation. Pharmabiotics in clinical and animal studies demonstrated multiple beneficial effects, indicating that targeting the gut microbiota is an intriguing therapeutic target for metabolic disorders (Okubo et al., 2018).

*Butyricimonas* is a Gram-negative anaerobic bacterial genus of the family *Odoribacteraceae*. They are present in the intestinal tract of several mammals, including rat and human (Sakamoto et al., 2009, 2014), but few species have been isolated (*B. faecihominis, B. synergistica, B. paravirosa*, *B. virosa*, and *B. phoceensis*). Treatment of metabolic disorders with metformin and statins significantly increased the relative abundance of *Butyricimonas* spp. in the gut, which was significantly correlated with metabolic parameters (Lee et al., 2018; Kim et al., 2019). However, the direct effect of *Butyricimonas* on metabolic improvements, and the underlying physiological mechanisms, have not been investigated.

The characteristics of *Butyricimonas* and our previous results support the hypothesis that *Butyricimonas* species are involved in commensal homeostasis between the gut microbiota and host, and exert a beneficial effect on host energy metabolism. To test this hypothesis, we investigated the metabolic effects induced by oral administration of live and heat-killed *Butyricimonas virosa* to mice with high-fat diet (HFD)-induced obesity. Furthermore, we conducted comparative analysis of metabolism-related transcripts.

## Materials and Methods

### Butyricimonas

*Butyricimonas virosa* (KCTC 15148) was purchased from the Korean Collection for Type Cultures (KCTC) and cultured in Columbia broth supplemented with 5% horse serum under anaerobic conditions (i.e., in an anaerobic jar) at 37°C for 5 days. Cultured *B. virosa* (estimated 5 × 10^8^ CFU/ml optical density) was washed in phosphate-buffered saline (PBS) and stored at −70°C until inoculation, and their viability was confirmed using the spread plate method. In addition, heat-killed *B. virosa* were prepared by autoclaving for 15 min at 121°C and 15 lb.

### Animal Model

Male 4-week-old C57BL/6 N mice were purchased from Samtako Co., Ltd. (Osan, South Korea) and acclimated to laboratory conditions for 1 week, during which the animals were housed in a temperature and humidity-controlled animal facility under a 12 h light–dark cycle at 22 ± 2°C and 55 ± 5% humidity, with *ad libitum* access to water and food. Mice were fed a 60% kcal HFD (FeedLab, Inc., Guri, South Korea) to induce metabolic disorders—such as obesity, hyperglycemia, and hyperlipidemia—for 16 weeks. The mice were treated with *B. virosa* by oral gavage at 1 × 10^8^ CFU/200 μl (HFD-Bu: live *B. virosa* during HFD, *n* = 6, or HFD-hk-Bu: heat-killed *B. virosa* during HFD, *n* = 6) daily for the final 6 weeks of HFD feeding. A regular diet (RD, 10% kcal; Purina Korea, Inc., Seoul, South Korea)-fed group (*n* = 6) and HFD-fed group (HFD without *B. virosa*, *n* = 5) were included as normal and disease controls, respectively.

### Metabolic Analysis

Body weight, serum glucose level, and food intake were recorded weekly. The serum glucose level was determined using the Accu-Chek Performa system (Roche Diagnostics, Mannheim, Germany) following fasting for 12 h (dark cycle). Intraperitoneal glucose tolerance testing (IPGTT) was performed 6 weeks after treatment with *B. virosa*. The mice were intraperitoneally injected with glucose solution (2 g/kg in PBS) after fasting 12 h (dark cycle), and the glucose level was measured by tail vein sampling using blood lancet at 15, 30, 60, and 120 min after injection. The incremental area under the curve (iAUC) for glucose was calculated for comparison of IPGTT results among time points. The Homeostatic Model Assessment of Insulin Resistance (HOMA-IR) index was used to evaluate insulin resistance (Matthews et al., 1985). Blood samples were collected by cardiac puncture and centrifuged at 10,000 rpm for 5 min to isolate serum. The serum levels of apolipoprotein B (ApoB), low-density lipoprotein (LDL), high-density lipoprotein (HDL), aspartate aminotransferase (AST), and alanine aminotransferase (ALT) were determined using a biochemical analyzer (AU480; Beckman Coulter, Brea, CA).

### Liver Histology

Hepatic tissue was fixed in 10% neutral formalin. Tissue samples were filtered and embedded in paraffin, cut into 4-μm sections, and stained with hematoxylin and eosin (H&E). Sections were observed using a microscope (Nikon, Tokyo, Japan), and micrographs were taken at ×100 magnification. The percentage of steatosis in images was assessed using ImageJ software (Bethesda, MD).

### Transcriptome Analysis

Ileum and liver tissues were immediately frozen in liquid nitrogen and stored at −70°C for later analysis of transcript levels. Total RNA was extracted using the RiboEx^™^ Kit (GeneAll, Seoul, South Korea). cDNA was synthesized using HyperScript^™^ RT Premix (GeneAll) according to the manufacturer’s instructions. SYBR Green PCR Master Mix and the StepOnePlus^™^ Real-Time PCR System (Applied Biosystems, Waltham, MA) were used to quantify mRNA levels. The following primer sets were used as: insulin receptor substrate (IRS)-1 (forward: 5′-TCCTATCCCGAAGAGGGTCT-3′; reverse: 5′-TGGGCATATAGCCATCATCA-3′), IRS-2 (forward: 5′-TCCAGAACGGCCTCAACTAT-3′; reverse: 5′-AGTGATGGGACAGGAAGTCG-3′), glucagon-like peptide-1 receptor (GLP-1R; forward: 5′-TCAGAGACGGTGCAGAAATG-3′; reverse: 5′-CAGCTGACATTCACGAAGGA-3′), Dipeptidyl peptidase-4 (DPP4; forward: 5′-TTGTGGATAGCAAGCGAGTTG-3′; reverse: 5′-CACAGCTATTCCGCACTTGAA-3′), zonula occludens-1 (ZO-1; forward: 5′-GCTCATAGTTCAACACAGCCTCCAG-3′; reverse: 5′-TTCTTCCACAGCTGAAGGACTCACAG-3′), peroxisome proliferator-activated receptor α (PPARα, forward: 5′-TCGGCGAACTATTCGGCTG-3′; reverse: 5′-GCACTTGTGAAAACGGCAGT-3′), PPAR𝛾 (forward: 5′-TGTGGGGATAAAGCATCAGGC-3′; reverse: 5′-CCGGCAGTTAAGATCACACCTAT-3′), Toll-like receptor 5 (TLR5; forward: 5′-AAGTTCCGGGGAATCTGTTT-3′; reverse: 5′-GCATAGCCTGAGCCTGTTTC-3′), and glyceraldehyde 3-phosphate dehydrogenase (GAPDH; forward: 5′-AACTTTGGCATTGTGGAAGG-3′; reverse: 5′-ACACATTGGGGGTAGGAACA-3′).

### Relative Abundance of *Butyricimonas*

The relative abundance of *Butyricimonas* in fecal samples was evaluated using SYBR^®^ Green PCR Master Mix and the StepOnePlus^™^ Real-Time PCR System (Applied Biosystems). Fecal samples were collected 6 weeks after treatment with *B. virosa*. Total DNA was extracted using the PowerSoil DNA Isolation Kit (MO BIO Laboratories Inc., Carlsbad, CA) according to the manufacturer’s instructions. Primer sets for total bacteria (515f, forward: 5′-GTGCCAGCMGCCGCGGTAA-3′; 806r, reverse: 5′-GGACTACHVHHHTWTCTAAT-3′) and *Butyricimonas* (Buty1f, forward: 5′-GGTGAGTAACACGTGTGCAAC-3′; Buty1r, reverse: 5′-TACCCCGCCAACTACCTAATG-3′) were used for amplification.

### Butyrate Analysis

Butyrate in feces was quantified using a modified version of a method published elsewhere (Cuervo et al., 2013; Jang et al., 2020). Homogenized mouse feces (10 mg/50 μl of PBS) was dried in a dry oven at 60°C to remove water. The dried sample was extracted with 500 μl of MeOH for 10 min in a shaking incubator. An internal standard, 4-methylvaleric acid (Sigma-Aldrich, St. Louis, MO), was added to the supernatant. The SCFA concentration was calculated using standard reagents (acetate, propionate, and butyrate; Sigma-Aldrich) with Autochro-3,000 software and the YL6100 GC system, which was equipped with a flame ionization detector (FID) and capillary column (DB-Wax column, 30 m × 0.25 mm × 0.25 μm; Agilent Technology, Santa Clara, CA). The inlet and detector temperatures were set to 220°C. Samples (2 μl) were introduced by splitless injection. The oven temperature was initially maintained at 50°C for 1 min and then increased to 220°C at a rate of 10°C/min.

### Western Blotting

Total liver protein was extracted using RIPA lysis buffer (GenDEPOT, Katy, TX) supplemented with a protease inhibitor cocktail solution (GenDEPOT). The homogenate was incubated at 4°C for 2 h. The protein concentration was measured by the Bradford method. Protein samples (20 μg) were subjected to 10% SDS-polyacrylamide gel electrophoresis and transferred to nitrocellulose membranes. The membranes were blocked with 5% bovine serum albumin (BSA) in Tris-buffered saline containing 0.1% Tween-20 (TBST) for 2 h. They were then incubated at 4°C overnight with the primary anti-GAPDH antibody and anti-GLP-1R polyclonal antibody (1:1,000; Abcam, Cambridge, UK). After incubation with an anti-rabbit IgG horse radish peroxidase (HRP)-conjugated antibody (1:5,000; GenDEPOT) for 1 h, bands were detected using a chemiluminescent peroxidase substrate (ECL Plus; GenDEPOT) and imaged using the ChemiDoc XRS System (Bio-Rad, Hercules, CA).

### Statistical Analysis

Data are presented as means ± standard error of mean (SEM). To quantify the *in vivo* mRNA levels relative to the internal control (GAPDH), the 2^–ΔΔCt^ relative quantification method (ΔΔCt = (C_t.Target_ − C_t.GAPDH_)_Group1_ − (C_t.Target_ − C_t.GAPDH_)_Group2_) was used. Statistical significance was assessed by Kruskal-Wallis H test, followed by Duncan’s *post-hoc* test. Statistical analysis was performed using RStudio (R Development Core Team, Vienna, Austria). A *p*-value of <0.05 was considered indicative of statistical significance.

## Results

### Effect of *B. virosa* on Metabolic Disorders

**Figure 1:**
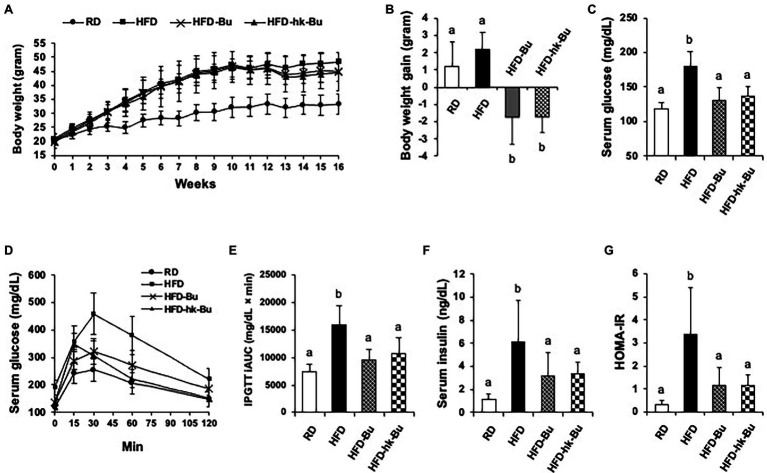
Effect of *Butyricimonas virosa* treatment on body weight **(A, B)**, fasting serum glucose level **(C-E)**, and insulin resistance **(F, G)**. Five-week-old C57BL/6 N mice were fed a HFD (60% lipid) for 16 weeks to induce metabolic disorders. Then, live and heat-killed *B. virosa* were orally administered daily for the final 6 weeks of HFD feeding. Body weight change was measured at 6 weeks after treatment with *B. virosa*. Treatment with *B. virosa* significantly improved metabolic profiles. RD: Regular diet (*N* = 6); HFD: High-fat diet (*N* = 5); HFD-Bu: Live *B. virosa* administration during HFD feeding (*N* = 6); and HFD-hk-Bu: Heat-killed *B. virosa* administration during HFD feeding (*N* = 6). Different superscript letters indicate significant differences (*p* < 0.05) according to Duncan’s *post-hoc* test.

Feeding the mice an HFD for 16 weeks induced weight gain; the average body weight of mice in the HFD group (46.5 ± 0.5 g) was significantly increased compared to that of mice in the RD group (32.0 ± 3.6 g). After treatment with *B. virosa* for 6 weeks, the body weight of mice in the HFD-Bu (44.7 ± 3.6 g) and HFD-hk-Bu (43.7 ± 5.7 g) groups was decreased compared to mice in the HFD group (48.2 ± 3.6 g; Figure 1A). The mice in the HFD group gained 2.2 ± 1.2 g after the 6-week HFD, whereas those in the HFD-Bu and HFD-hk-Bu groups lost 2.2 ± 1.7 and 2.0 ± 0.8 g, respectively (Figure 1B). Daily food intake was not significantly different among the groups.

Treatment with *B. virosa* significantly improved serum glucose regulation. *B. virosa* significantly reduced the HFD-induced increase in fasting serum glucose levels in mice in the HFD-Bu (131.2 ± 17.6 mg/dl) and HFD-hk-Bu (137.1 ± 13.5 mg/dl) groups compared to the HFD group (180.2 ± 21.3 mg/dl; Figure 1C) after 6 weeks of *B. virosa* treatment and significantly ameliorated glucose tolerance (IPGTT; Figures 1D,E). Moreover, *B. virosa* reduced the serum insulin level in the HFD-Bu (3.2 ± 2.0 ng/dl) and HFD-hk-Bu (3.4 ± 1.0 ng/dl) groups compared to the HFD group (6.1 ± 3.6 ng/dl) and significantly improved the HOMA-IR scores (HFD: 3.4 ± 2.0, HFD-Bu: 1.1 ± 0.8 and HFD-hk-Bu: 1.2 ± 0.4; Figures 1F,G).

**Figure 2:**
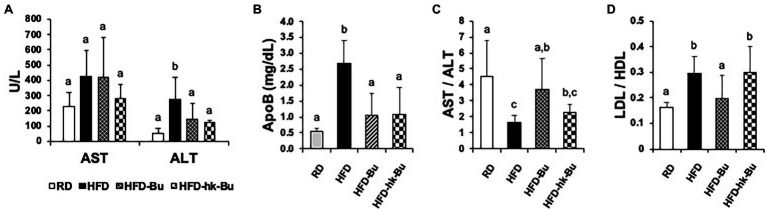
Biochemical analysis of serum. Significant recovery of the serum ALT **(A)** and ApoB **(B)** levels was observed in both the HFD-Bu and HFD-hk-Bu groups. The AST/AST **(C)** and LDL/HDL **(D)** ratios were significantly recovered only in the HFD-Bu group. Blood samples were collected *via* cardiac puncture, biochemical analysis was performed using a biochemical analyzer. Different superscript letters indicate significant differences (*p* < 0.05) according to Duncan’s *post-hoc* test. ALT, Alanine aminotransferase; ApoB, Apolipoprotein B, LDL, HFD-Bu, Live *B. virosa* administration during HFD feeding; HFD-hk-Bu, Heat-killed *B. virosa* administration during HFD feeding; Low-density lipoprotein HDL, High-density lipoprotein; and AST, Aspartate aminotransferase.

A significant decrease in the HFD-induced increased ALT and ApoB levels was observed in the HFD-Bu and HFD-hk-Bu groups compared to the HFD group, but the AST/ALT ratio was significantly improved only in the HFD-Bu group (Figures 2A–C). In addition, the LDL/HDL ratio was significantly decreased only in the HFD-Bu group (Figure 2D). Daily food intake was not significantly different among the groups.

### Effect of *B. virosa* on Liver Histology

**Figure 3:**
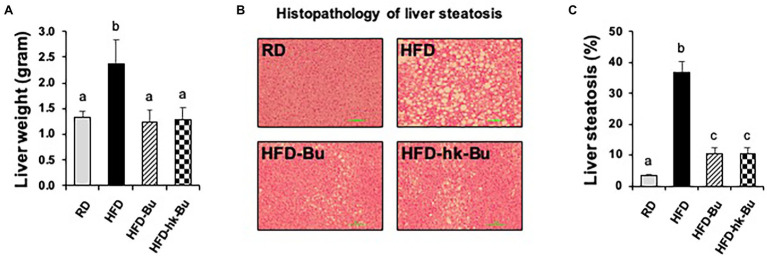
Liver histology. C57BL/6 N mice fed a HFD (60% lipid) for 16 weeks showed an increase in the total liver weight **(A)** and lipid deposition in the liver **(B)**. Both treatment with live and heat-killed *Butyricimonas virosa* for 6 weeks significantly reduced the liver weight and lipid deposition. **(C)** Percentage of steatosis in liver histology images was analyzed with ImageJ software. Histological analysis of liver steatosis was performed using H&E staining. Different superscript letters indicate significant differences (*p* < 0.05) according to Duncan’s *post-hoc* test. HFD, High-fat diet; H&E, Hematoxylin and eosin.

The HFD-induced increased liver weight was significantly decreased by *B. virosa* treatment compared to the HFD group (Figure 3A). The severe liver steatosis in the HFD group was recovered by both live and heat-killed *B. virosa* (Figures 3B,C).

### Quantification of *B. virosa* and Butyrate in Feces

**Figure 4:**
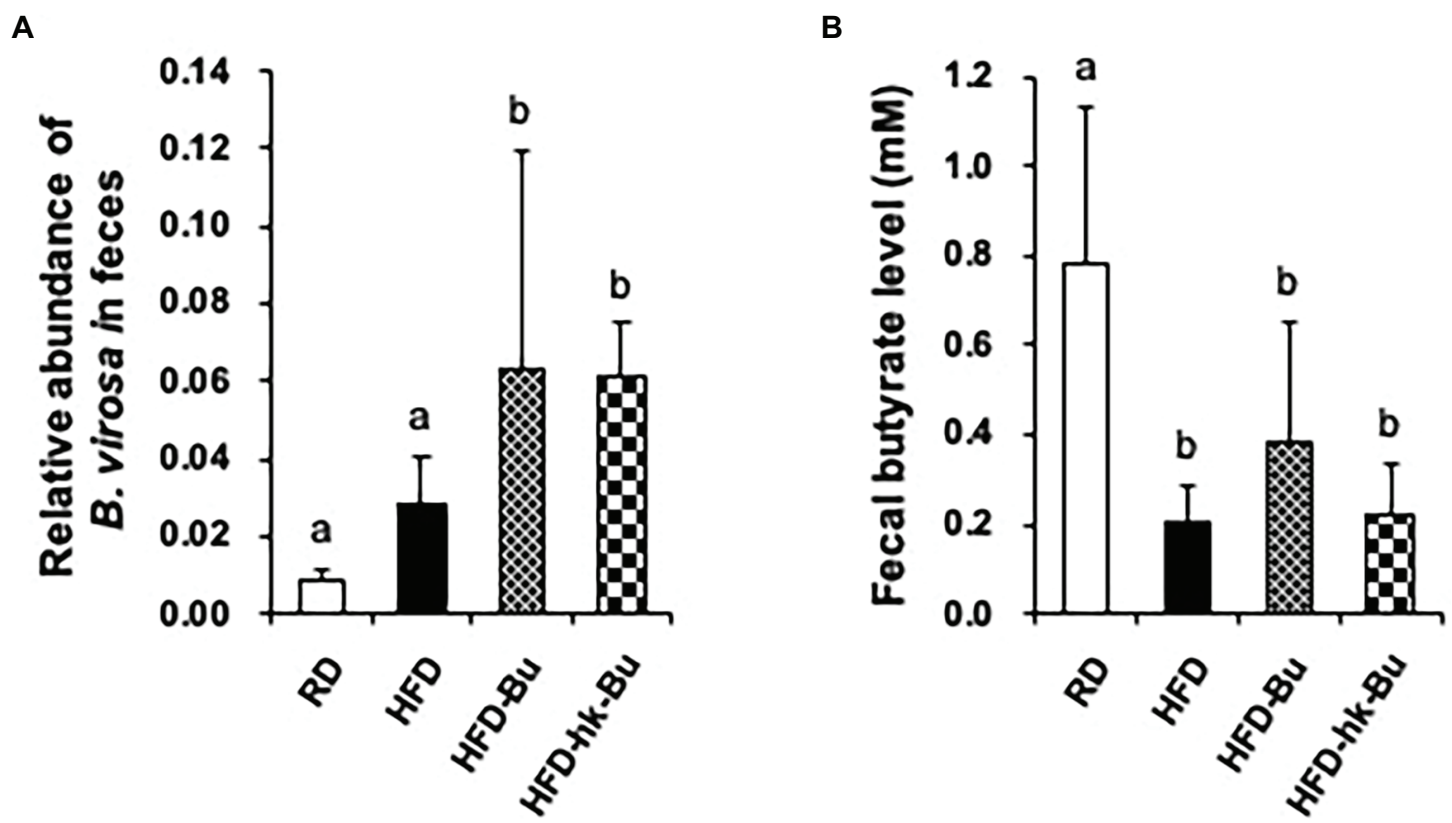
Quantification of *Butyricimonas virosa* and butyrate levels in feces. Fecal samples were collected at 6 weeks after treatment with *B. virosa*. Relative abundance of *B. virosa* analyzed using quantitative PCR **(A)**, the concentration of butyrate was measured using gas chromatography **(B)**. The relative abundance of *B. virosa* and the concentration of butyrate in fecal samples were significantly increased after treatment of *B. virosa* compared to HFD group.

The relative abundance of *B. virosa* was 6.3 ± 5.7% and 6.2 ± 1.3% in fecal samples from mice in the HFD-Bu and HFD-hk-Bu groups, respectively, which were significantly higher than in the HFD group (2.8 ± 1.2%; Figure 4A). Furthermore, the concentration of butyrate in fecal samples was significantly decreased in the HFD group (0.21 ± 0.08 mM) compared to the RD group (0.78 ± 0.35 mM; Figure 4B). The difference in concentration of butyrate between HFD and HFD-Bu groups was not statistically significant (Figure 4B). The difference in the levels of acetate and propionate between groups were also not significant (data not shown).

### Transcriptome Analysis in the Liver and Ileum

**Figure 5:**
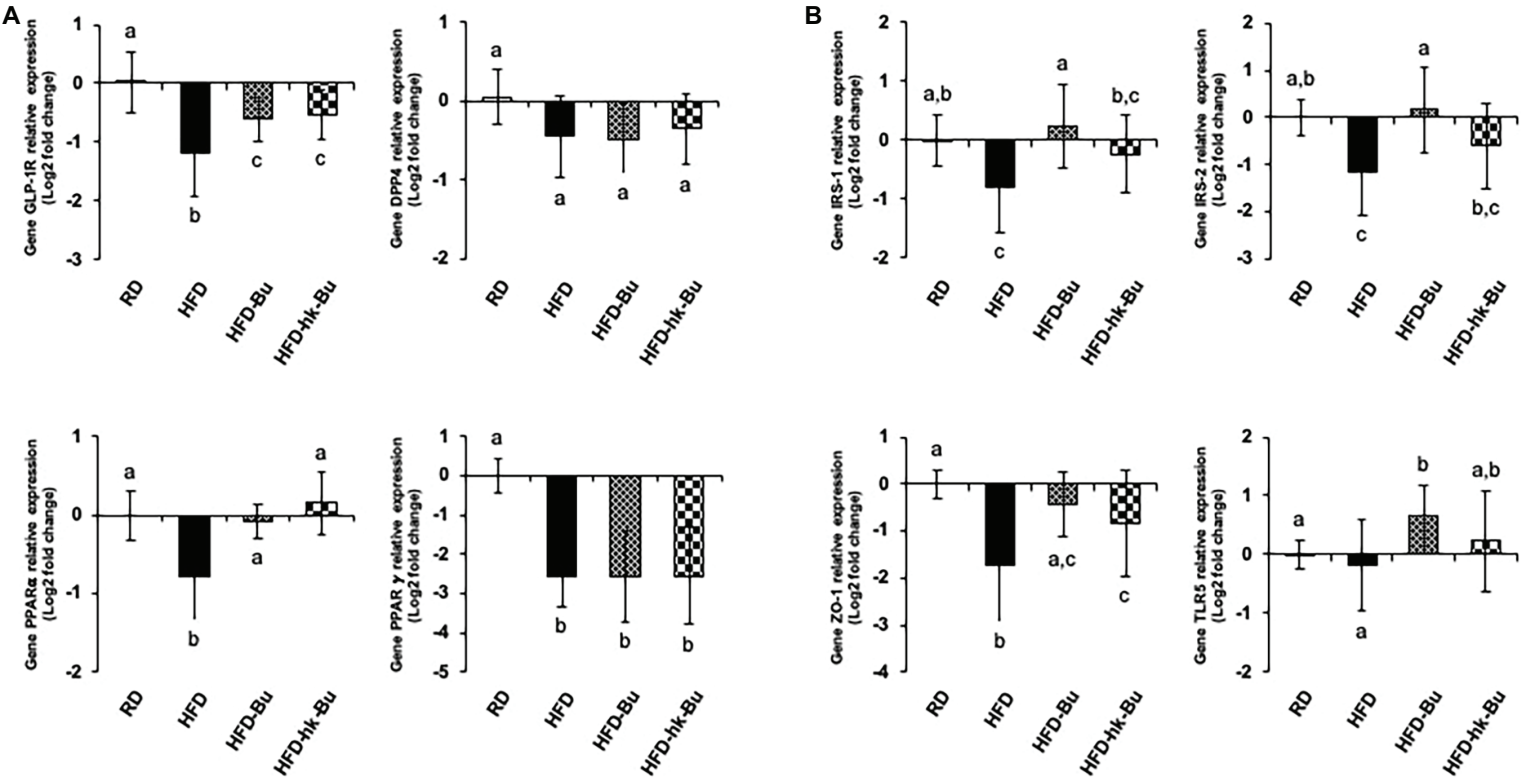
Transcriptome analysis in the liver **(A)** and ileum **(B)**. Relative levels of mRNA associated with glucose regulation and lipid metabolism measured using quantitative PCR. The 2^−ΔΔCT^ relative quantification method was used for analysis of the level of relative mRNA expression (log2 fold change) compared to that of GAPDH expression as an internal control. Different superscript letters indicate significant differences (*p* < 0.05) according to Duncan’s *post-hoc* test. GAPDH, Glyceraldehyde 3-phosphate dehydrogenase.

HFD significantly downregulated the levels of GLP-1R (0.50 ± 0.23) and PPARα (0.62 ± 0.24) in the liver compared with RD (GLP-1R: 1.08 ± 0.38 and PPARα: 1.02 ± 0.24; Figure 5A). Treatment with *B. virosa* significantly upregulated the liver levels of GLP-1R in the HFD-Bu (0.67 ± 0.17) and HFD-hk-Bu (0.72 ± 0.22) groups, and of PPARα in the HFD-Bu (0.96 ± 0.14) and HFD-hk-Bu (1.15 ± 0.32) groups, compared to the HFD group (Figure 5A). The levels of DPP4 and PPAR𝛾 were not significantly different between the *B. virosa*-treated and HFD groups.

In the ileum, HFD significantly downregulated the levels of IRS-1 (0.64 ± 0.27), IRS-2 (0.54 ± 0.33), and ZO-1 (0.41 ± 0.31) compared with RD (IRS-1: 1.04 ± 0.30, IRS-2: 1.03 ± 0.29, and ZO-1: 1.02 ± 0.21), whereas the level of TLR5 was not significantly different between the RD (1.01 ± 0.17) and HFD (1.03 ± 0.64) groups (Figure 5B). Furthermore, *B. virosa* significantly upregulated the levels of IRS-1 in the HFD-Bu (1.35 ± 0.89) and HFD-hk-Bu (0.94 ± 0.47) groups, and those of IRS-2 in the HFD-Bu (1.32 ± 0.74) and HFD-hk-Bu (0.78 ± 0.42) groups, compared to the HFD group (Figure 5B). Moreover, *B. virosa* significantly upregulated the level of ZO-1 in the HFD-Bu (0.81 ± 0.34) and HFD-hk-Bu (0.71 ± 0.46) groups, and of TLR5 in the HFD-Bu (1.67 ± 0.68) and HFD-hk-Bu (1.37 ± 0.88) groups, compared to the HFD group (Figure 5B).

### Regulation of GLP-1R in the Liver by *B. virosa*

**Figure 6:**
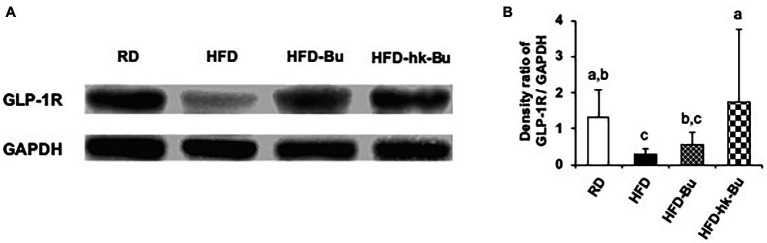
GLP-1R protein expression in the liver. Live and heat-killed *Butyricimonas virosa* were administered daily for the final 6 weeks of HFD feeding. Total protein extracted from liver lysates was used in Western blot GLP-1R analysis **(A)**. GLP-1R protein was detected using anti-GLP-1R polyclonal antibodies; the expression ratio was quantified compared to the expression of GAPDH detected using anti-GAPDH polyclonal antibodies **(B)**. Different superscript letters indicate significant differences (*p* < 0.05) according to Duncan’s *post-hoc* test. GLP-1R, Glucagon-like peptide-1 receptor; HFD, High-fat diet; GAPDH, Glyceraldehyde 3-phosphate dehydrogenase.

The HFD-induced downregulation of the GLP-1R mRNA level in the liver was increased by heat-killed *B. virosa* (Figure 5A). In addition, the GLP-1R protein level in the liver, which was significantly decreased by the HFD, was upregulated by *B. virosa* compared to the HFD group (Figures 6A,B).

## Discussion

*Butyricimonas virosa* had a beneficial effect on metabolic disorders, including obesity and hyperglycemia, *via* GLP-1R in the liver in a mouse model of obesity. This finding is in agreement with a recent report that the abundance of *Butyricimonas* spp. is related to metabolic improvements (Lee et al., 2018; Kim et al., 2019). In addition, enrichment of *B. virosa* was observed in mice colonized with the microbiota of lean twins discordant for obesity (Ridaura et al., 2013). In a human study, a high level of the genus *Butyricimonas* was associated with significantly decreased triglyceride levels and body mass index (BMI; Fu et al., 2015). Interestingly, heat-killed *B. virosa* significantly ameliorated body weight gain, hyperglycemia, insulin resistance, and liver steatosis similarly to live *B. virosa*. Therefore, we considered that the metabolic improvements were not mediated by *B. virosa*-produced butyrate.

In this study, an increase in the relative abundance of *B. virosa* was identified in the fecal samples of mice in the HFD-Bu and HFD-hk-Bu groups but was significant only in the former group. We predicted that metabolic improvements would not be observed in the HFD-hk-Bu group. *Butyricimonas* is a butyrate-producing bacterial genus and SCFAs, such as acetate, propionate, and butyrate, regulate the epithelial expression of genes involved in energy metabolism (Den Besten et al., 2013). Moreover, the administration of butyrate improved metabolic disorders, including hyperglycemia and hepatic lipogenesis, in a manner mediated by AMPK, GLUT4, and GLP-1 (Gao et al., 2019; Zhang et al., 2019).

Previous studies on *A. muciniphila* support the effect of heat-killed *B. virosa* on metabolic improvements. Live and pasteurized *A. muciniphila* at 70°C improved the metabolism of rodents and humans with obesity and diabetes (Depommier et al., 2019, 2020). Although it is unclear why heat-killed *B. virosa* induced metabolic improvements in this study, there are several plausible mechanisms. First, the effect of *B. virosa* may be mediated by a protein stable to autoclaving. Certain active bacterial components that mitigate insulin resistance and glucose tolerance are unaffected by heat treatment. Moreover, heat-killed *B. virosa* may modulate the gut microbiota and metabolic improvements. Although the effect of *B. virosa* on the gut microbiota was not investigated in this study, bacterial components and metabolites can act as prebiotics, modulating the gut microbiota (Salminen et al., 2021). Live and heat-killed *B. virosa* may have different modes of action, on which further studies are needed.

GLP-1R was implicated in glucose homeostasis following *B. virosa* treatment. Activation of GLP-1R is involved in regulation of hyperglycemia, and GLP-1R agonists including exenatide, liraglutide, and lixisenatide are important therapeutics for type II diabetes (Burcelin et al., 2014) Especially, GLP-1 secreted from intestinal cells activates glucose-dependent insulin secretion and inhibits glucagon release (MacDonald et al., 2002). SCFAs increase the secretion of GLP-1 and GLP-1R (Tolhurst et al., 2012; Zhang et al., 2019). Therefore, we hypothesized that the metabolic improvements induced by *Butyricimonas* were caused by an increase in its SCFA production. However, the expression of GLP-1R in the intestine was not significantly increased by *Butyricimonas* (data not shown). Interestingly, GLP-1R was upregulated in the liver, in which its activation is implicated in glucose regulation by *Butyricimonas*. Indeed, GLP-1 is closely related to hepatocyte fatty acid β-oxidation and insulin sensitivity (Svegliati-Baroni et al., 2011), and GLP-1R in hepatocytes modulated insulin signaling, thereby ameliorating hepatic steatosis (Gupta et al., 2010). In this study, the hepatic steatosis induced by the HFD was improved by both live and heat-killed *B. virosa*; thus, *Butyricimonas* may modulate hepatic lipogenesis *via* GLP-1R activation.

In addition, DPP4, which inactivates GLP-1, is an important therapeutic target for hyperglycemia. DPP4 regulation was not observed in this study, so may not be involved in GLP-1R-mediated glucose regulation by *Butyricimonas*. Hepatic DPP4 is important in insulin resistance and the anti-diabetic effect of DPP4 inhibitors is mediated by upregulation of intact GLP-1 (Kim et al., 2016). However, an anti-glycemic effect of DPP4 inhibitors is apparent in mice lacking GLP-1R (Hansotia et al., 2004), and GLP-1R antagonism does not eliminate the anti-glycemic effect of DPP4 inhibitors in patients with T2D (Nauck et al., 2016). Moreover, GLP-1R agonists protect endogenous GLP-1 from degradation by DDP4 and are used in the treatment of T2D (Drucker et al., 2010).

PPARs are essential regulators of energy metabolism. Among them, PPARγ is a major therapeutic target for glucose regulation. Interestingly, the expression level of PPARα was significantly increased, instead of that of PPARγ. The role of PPARα in modulating glucose homeostasis is unclear. PPARα-null mice were protected against HFD-induced insulin resistance (Guerre-Millo et al., 2001), and PPARα deficiency reduced insulin resistance in apoE-null mice (Tordjman et al., 2001). In contrast, the absence of PPARα increased hepatic glucose production from lactate and glycerol irrespective of the insulin level (Xu et al., 2002). Moreover, fibrates, which are selective PPARα activators, improved HFD-impaired insulin sensitivity, thereby suppressing the expression of carcinoembryonic antigen-related cell adhesion molecule 1 (CEACAM1) and promoting insulin clearance (Guerre-Millo et al., 2000; Ramakrishnan et al., 2016). PPARα regulates hepatic lipogenesis by modulating fatty acid uptake and oxidation (Pawlak et al., 2015). Insulin resistance is associated with hepatic steatosis and is a hallmark of non-alcoholic fatty liver (Chao et al., 2019). In this study, the HFD-induced increased liver weight and steatosis were significantly ameliorated by *B. virosa* treatment compared to the HFD group. Therefore, hepatic glucose regulation may be affected by fatty acid homeostasis *via* PPARα in the liver.

HFD-induced dysbiosis of the gut microbiota affects the expression of various genes related to energy metabolism, inflammation, and lipid profiles (Boulange et al., 2016; Teixeira et al., 2021). In this study, live *B. virosa* ameliorated the HFD-induced downregulation of mRNA levels of IRS-1, IRS-2, ZO-1, and TLR5 in the ileum, likely related to the activation of GLP-1R for glucose regulation in the liver. A lack of insulin receptors in the intestine contributes to the pathophysiological changes seen in patients with type II diabetes (Ussar et al., 2017). The intestinal epithelial barrier, which is related to dysbiosis of the gut microbiota, affects liver function and insulin resistance (Damms-Machado et al., 2017). ZO-1 is an important intracellular tight junction protein, whose disruption is linked to hyperglycemia (Thaiss et al., 2018). TLR5-deficient mice showed features of metabolic syndrome, including hyperglycemia and insulin resistance, which were significantly correlated with the altered gut microbiota (Vijay-Kumar et al., 2010).

Our findings suggest that *B. virosa* is implicated in glucose regulation in obese mice, but the absence of a mechanistic analysis was a limitation. We evaluated crosstalk between the gut microbiota and GLP-1R in the liver. Although *B. virosa* upregulated several mRNA transcripts in the intestine, we did not analyze downstream effects of GLP-1R activation in the liver. In addition, to generalize the anti-hyperglycemic effect of *Butyricimonas*, intervention studies using other animal models, including a non-obese diabetic mouse, are needed. Furthermore, investigation of *Butyricimonas*-induced changes in the gut microbiota under a variety of conditions would provide insight into the effect of *Butyricimonas* on glucose regulation.

## Conclusion

Treatment with live and heat-killed *B. virosa* ameliorated changes in body weight, serum glucose level, insulin resistance, and liver steatosis in a mouse model of HFD-induced obesity. Moreover, GLP-1R in the liver was activated, which may be linked to in the metabolic improvements induced by *Butyricimonas*. Our results provide a rationale for the development of pharmabiotics based on *Butyricimonas* spp. for the prevention and treatment of type II diabetes.

## Data Availability Statement

The original contributions presented in the study are included in the article/supplementary material, further inquiries can be directed to the corresponding author.

## Ethics Statement

The animal study was reviewed and approved by Institutional Animal Care and Use Committee (IACUC) of Sahmyook University (SYUIACUC 2019-004).

## Author Contributions

HL and KK: concept and design. JA, JK, DC, and HK: mouse model. HL, JA, JK, and DC: analysis or interpretation of data. HL: drafting of the manuscript. YS, CKL, HK, SBK, and KK: critical revision of the manuscript. All authors contributed to the article and approved the submitted version.

## Conflict of Interest

The authors declare that the research was conducted in the absence of any commercial or financial relationships that could be construed as a potential conflict of interest.

## Publisher’s Note

All claims expressed in this article are solely those of the authors and do not necessarily represent those of their affiliated organizations, or those of the publisher, the editors and the reviewers. Any product that may be evaluated in this article, or claim that may be made by its manufacturer, is not guaranteed or endorsed by the publisher.
